# Joint Relay Selection and Power Allocation through a Genetic Algorithm for Secure Cooperative Cognitive Radio Networks

**DOI:** 10.3390/s18113934

**Published:** 2018-11-14

**Authors:** Md Arifur Rahman, YoungDoo Lee, Insoo Koo

**Affiliations:** School of Electrical Engineering, University of Ulsan, 93-Daehak-ro, Namgu, Ulsan 44610, Korea; rassel.aece@gmail.com (M.A.R.); leeyd1004@naver.com (Y.L.)

**Keywords:** cooperative communication, mixed integer programming, genetic algorithm, secrecy rate, multi-relay selection, power allocation

## Abstract

In cooperative cognitive radio networks (CCRNs), there has been growing demand of transmitting secondary user (SU) source information secretly to the corresponding SU destination with the aid of cooperative SU relays. Efficient power allocation (PA) among SU relays and multi-relay selection (MRS) are a critical problem for operating such networks whereas the interference to the primary user receiver is being kept below a tolerable level and the transmission power requirements of the secondary users are being satisfied. Subsequently, in the paper, we develop the problem to solve the optimal solution for PA and MRS in a collaborative amplify-and-forward-based CCRNs, in terms of maximizing the secrecy rate (SR) of the networks. It is found that the problem is a mixed integer programming problem and difficult to be solved. To cope with this difficulty, we propose a meta-heuristic genetic algorithm-based MRS and PA scheme to maximize the SR of the networks while satisfying transmission power and the interference requirements of the networks. Our simulation results reveal that the proposed scheme achieves near-optimal SR performance, compared to the exhaustive search scheme, and provides a significant SR improvement when compared with some conventional relay selection schemes with equal power allocation.

## 1. Introduction

Cognitive radio networks (CRNs) [1] generally consists of primary users (PUs) and secondary users (SUs), which can potentially solve the spectrum scarcity problem. In CRNs, the SUs opportunistically access the spectrum and utilize the spectrum for their own data transmission. The idea of improving radio resources and the data rate has recently been attracting researchers and industries. Cognitive radio (CR) effectively uses the radio resource by adopting an interweave, overlay and underlay spectrum access (SA) techniques [2]. Among them the interweave SA technique does not allow the SUs to utilize the PUs spectrum. In the overlay SA technique, SUs opportunistically use the spectrum resources while the PUs are not using them. Whereas, in the underlay SA technique, the SUs are allowed to transmit their own data simultaneously despite the presence of the PUs until the interference induced by the SUs transmissions remain bellow an acceptable level (denoted as interference threshold) of the PU receiver. CR and cooperative communications (CC) may provide smart solutions for efficient utilization of spectrum resources. In CC, the source use a cooperative node to transmit source information to the intended destination. It can also decrease the interference by subsiding the transmission power of the source. In cooperative cognitive radio networks (CCRNs), the SU source needs to send information secretly to the SU destination with the aid of cooperative SUs relay. The objective of physical layer security in wireless communications is to enable secure communications between the users by exploiting the physical properties of the wireless communication channel in order to calculate the amount of secure information at the receiver. In cognitive radio networks (CRNs), a secondary user selection scheme to enhance the security of the primary user was studied by Qin et al. [3] in which minimal interference-based and maximal jamming-based schemes are proposed to improve the security of the primary user. Similarly, power allocation policy for CRNs with the convex combination of the upper and the lower transmission power of the secondary transmitter was proposed in [4] where the closed-form expression for the outage probability is derived to analyze the performance of the networks. In an addition, secrecy analysis of random multiple-input and multiple-output (MIMO) wireless networks, uplink secrecy capacity in device-to-device (D2D)-enabled cellular networks, physical layer security of hybrid milimeter wave networks were also studied in [5,6,7] to enhance the overall security of the networks. Wireless social networks and its recent advancement with applications and challenges are jointly studied in [8] where some security aspects of the networks along with some future research directions are present to enhance the security of the networks.

In CCRNs, relay cooperation not only reduces the required transmission power of the SUs, but it can also improve the physical layer security of the networks. Cooperative relaying is a very useful technique to improve the secrecy rate (SR) of cooperative wireless sensor networks (CWSNs), and this has been widely studied in many works [9,10,11,12,13,14] in which the SR of the networks is maximized through optimal relay selection (ORS) and optimal power allocation (OPA). However, exhaustive search (ES) and conventional optimization techniques (COTs) are used to solve the ORS and the OPA problems to maximize the SR of the networks. To improve the SR of CCRNs with the aid of the cooperative relaying concept, some research work has already investigated how to solve the problems of SU relay selection (RS), power allocation (PA) of the SU source and relay, and bandwidth allocation [15,16,17,18,19] by using ES and COTs. As is known, searching optimal solution for CCRNs is usually very expensive, so ES and COTs for multi-relay selection (MRS) and PA are not efficient approaches to maximize the SR of the networks. However, using a closed form solution for MRS and PA to SU relays is not valid, and needs frequent updates of the solution because of the time-varying wireless medium. In addition, COTs cause high complexity because the computational load is very high which causes undesired delays in updating the optimal solutions. To solve the complexity problem of COTs, a low-complexity timer-based MRS and PA were introduced by Rahman et al. [20] where the throughput of the networks is maximized by using handshake-based MRS and PA algorithms. However, the secrecy rate maximization of CCRNs was not considered in the work. Therefore, in this paper, we mainly focus on solving the MRS and PA problems to maximize the SR of the networks to block an eavesdropping attack at the physical layer of the networks. Due to considering a joint optimization problem, the objective function becomes a mixed integer programming problem (MIP) and is difficult to solve due to the coexistence of binary integer and real valued variables in our problem. When the objective function is too complicated to be mathematically analyzed, applying meta-heuristic genetic algorithm (GA) seems rational.

The GA has been well known to be an effective search approach that utilizes genetic evolution process to generate new chromosome to efficiently search for the optimal solution. Therefore, in this paper, we focus on solving SU relays selection and the PA problems to maximize the secrecy rate of the networks by applying low-complexity meta-heuristic GA. To solve the problem, GA is applied in this paper has two noticeable features. Firstly, the chromosome of the GA-based scheme is comprised of a binary integer string and one real number string to solve the SU relays selection and the SU relays PA problems, respectively. Secondly, a combined crossover and mutation operations are needed to apply for accommodating these new chromosomes of the proposed scheme. Therefore, in this paper we study the MRS and PA problems for SR maximization of a collaborative AF-based CCRNs and further solve the problems by the GA-based MRS and PA scheme. The main contributions of this paper are summarized as follows:

- Different from the existing researches on physical layer security in both CWSNs and CCRNs such as [9,10,11,12,13,14,15,16,17,18,19,21,22,23,24,25,26] in which some COTs (due to their high computation load) are adopted while causing high complexity and ES (due to high computational time) as well as undesired delays to solve the optimization problem. Therefore, a low-complexity meta-heuristic GA-based scheme is proposed in this paper to solve the optimization problem.
- We develop a meta-heuristic GA to overcome the difficulty arising from the MIP problem due to the coexistence of binary integer and real valued variables in the problem.
- To comprehensively evaluate the SR performance of the proposed scheme, we compare the proposed scheme with opportunistic RS (ORS), partial RS (PRS), and random RS schemes according to the different node locations, numbers of SU relays, maximum permissible transmission power of the SUs, interference thresholds for the PU receiver, variances of the additive white Gaussian noise (AWGN).
- We propose a low complexity GA-based solution which can solve the optimization problem very efficiently with much lower computational complexity and shows a near optimal performance with ES scheme.
- We verify through simulation results that, the proposed scheme achieves highest SR performance than some other conventional schemes with a much lower computational complexity than the ES scheme.
- It is also shown that the computation time of the proposed scheme is effectively reduced until when the maximum SR of the considered CCRNs is archived.

The remainder of this paper is prepared as follows. Some related works are reviewed in Section 2. The system model and problem formulation are shown in Section 3. The proposed GA-based MRS and PA scheme is briefly described in Section 4. Simulation results and the computation complexity analysis of all compared schemes are presented in Section 5 and Section 6 concludes the paper with future research directions.

## 2. Related Works

The SR maximization problem is a well-researched topic for future wireless networks but it has not been deeply investigated in CWSNs and CCRNs.

### 2.1. SR Maximization of CWSNs and CCRNs

In CWSNs, the secrecy sum rate maximization of a MIMO networks through a relay node in the presence of a passive eavesdropper with analog network coding (ANC) was studied in [9]. Secure transmission approaches for several levels of eavesdropped channel state information (CSI) at the transmitter were evaluated by the authors. Similarly, two-phase distributed beamforming in a two-way relay network, and PA to enhance the secrecy sum rate of the data exchange were proposed in [10]. Three different schemes (namely, optimal beamforming, null-space beamforming, and artificial noise beamforming) were evaluated to show the performance of the SR. However, two of the schemes (with the concept of ANC limits only for CWSNs) do not ensure physical layer security for CCRNs. In CWSNs, energy efficiency and security issues in two-way relying concept was studied in [11] where the eavesdropping attack is being prevented while the legitimate users are transmitting their confidential information. The main objective of the work was to efficiently allocate transmission power and energy to the source and the relay to maximize the secure energy efficiency (EE) of the networks by satisfying the power constraint and the minimum target SR requirement of the networks. Similarly, secure EE maximization for a collaborative AF-based relay networks in the presence of an eavesdropper was studied in [12], where the authors jointly solve the source and relay PA problems by satisfying the maximum permissible transmission power and minimum target SR requirement, respectively. Energy-efficient secure communications over a decode-and-forward relay channel for CWSNs was studied [13] in which secure EE is maximized by satisfying some network constraints and a sub-optimal solution was also proposed to maximize the secure EE of the the networks. Similarly, secure communications in wireless relay networks with considering an eavesdropper was studied in [14] where an optimal PA strategy was proposed to maximize SR of the networks. Power-constrained SR maximization for joint relay and jammer selection in CWSNs was studied in [15] where an intermediate node is selected for data transmission while the others are used as friendly jammers to disrupt an eavesdropper by generating artificial noise. However, the above-mentioned schemes for SR maximization address only cooperative wireless networks [13,14,15]. Therefore, several works have been investigated to improve the SR of CCRNs by selecting the SU relay to forward the signal of the source by allocating optimal power to the SU relays.

In CCRNs, bandwidth efficient relaying technique was proposed by El-Malek et al. [16] in which two PA optimization problems were formulated to minimize the symbol error rate of the PUs and the SUs, along with maximizing the sum rate of the networks. A Lagrangian method [16] was used to solve the problems while satisfying the maximum permissible power budget constraint. However, an optimal solution for PA requires exhaustive search, which involves high computation complexity. Secure cooperative half-duplex cognitive radio networks through exhaustive search was adopted [17] where the *k*-th best relay is selected for PRS and ORS in order to maximize the SR of the networks. Similarly, RS for security enhancement in cognitive relay networks to demonstrate the performance of the secrecy outage probability was studied [18] where the first selected relay is considered for transmitting secrecy information to the destination and the second selected relay is used as a friendly jammer. A cooperative cognitive radio model for enhancing physical layer security in two-path AF relaying networks was studied in [19] where a Lagrangian multiplier method is formulated to obtain an OPA in order to maximize the SR of the system. In CWSNs, RA algorithm to maximize the SR of the networks was studied in [21] where an OPA is being obtained under satisfying transmission power constraints. To maximize the secrecy data rate, four different networks scenarios were studied with the assumption of imperfect CSI of the eavesdropper links. Secure CC scheme for orthogonal frequency-division multiple access CRNs was studied in [22] where a primary base station secret information is relayed to the distant PUs by the aid of the selected SUs relay while a set of passive eavesdropper coexists in the networks. A frame-based transmission system was considered where the secondary network is incentivized by the primary network if the SUs attend in cooperation with PUs to help some distance PUs to satisfy their SR requirement. Lagrange approach is used for solving the formulated optimization problem to maximize the SR of the PUs and SUs. Similarly, in CCRNs, guaranteed SR for the PUs was studied by Mokari et al. [23] where secondary transmitter secret information is transmitted to the secondary receiver in the presence of a set of SUs relay and the eavesdropper. To solve the non-convex optimization problem, decomposition is used to divide the main problem into three sub-problems and solve the problem efficiently to maximize the SR of the SUs. Nevertheless, the RA, RS, and PA problems of references [21,22,23] are being solved by COTs which require high computational complexity and undesired delays in updating the solutions of the optimization problem. Secrecy throughput maximization for multi-input-single-output CRNs in slow fading channels was studied in [24] where the secrecy throughput of the PU is maximized while satisfying the secrecy outage constraint at the PU and a throughput constraint at the SU. To maximize the secrecy throughput of the PU, adaptive and non-adaptive transmission strategies are proposed when CSI of the eavesdroppers are available. Similarly, cooperative transmission for securing a decode-and-forward two-hop network with the coexistence of multiple cooperative nodes and a potential eavesdropper was studied in [25] where an opportunistic relaying with artificial jamming secrecy scheme is proposed to maximize the ergodic SR of networks. Signal design and optimization techniques of enhancing wireless secrecy via cooperation were studied in [26] where signal processing perspective of physical layer security of cooperative system is overviewed and some future research directions are discussed for CWSNs.

### 2.2. GA for Resource Allocation in CWSNs and CCRNs

To the best of our knowledge, very little works have been done in CCRNs by using a GA. Okati et al. [27] studied a novel GA which can solve the resource allocation (RA) and cooperative node selection problems for maximizing the secrecy capacity of wireless communications networks. The RA and RS problems are jointly studied by Fang et al. [28] for maximizing the capacity of a cooperative wireless networks. To maximize the sum rate of orthogonal frequency division multiplexing-based CWSNs, a GA was proposed by Lai et al. [29] to solve the subcarrier pairing, PA, and RS problems of the system. A GA-based pilot allocation scheme for a massive MIMO system was studied by Zhang et al. [30] in which the sum rate is maximized under the proposed scheme. In CCRNs, a GA for MRS and PA for two-way relaying was studied by Ahmad et al. [31] in which the sum rate of the networks is maximized by satisfying the transmission power and interference requirements of the networks. In CCRNs, a GA-based scheme was jointly proposed by Yan et al. [32] in which a channel allocation and a cooperation set assignment is executed in such a way that for given time, the average transmission rate of the users achieve its maximal fairness. The physical layer security issues of CCRNs and PA method of the users were out of scope of their work. Therefore, GA-based scheme for SU relays selection and PA to the relays of CCRNs is very essential to study jointly to maximize the SR of the networks. In summary, existing works on RS and PA have generally considered COTs or ES to maximize the SR of the networks [9,10,11,12,13,14,15,16,17,18,19,21,22,23,24,25,26]. To the best of our knowledge, a low-complexity solution for MRS and PA for a collaborative AF-based CCRNs is still an open problem. Therefore, a GA-based scheme is proposed in this paper to select the SU relays and PA to the SU relays to maximize the SR of the networks.

## 3. System Model

**Figure 1:**
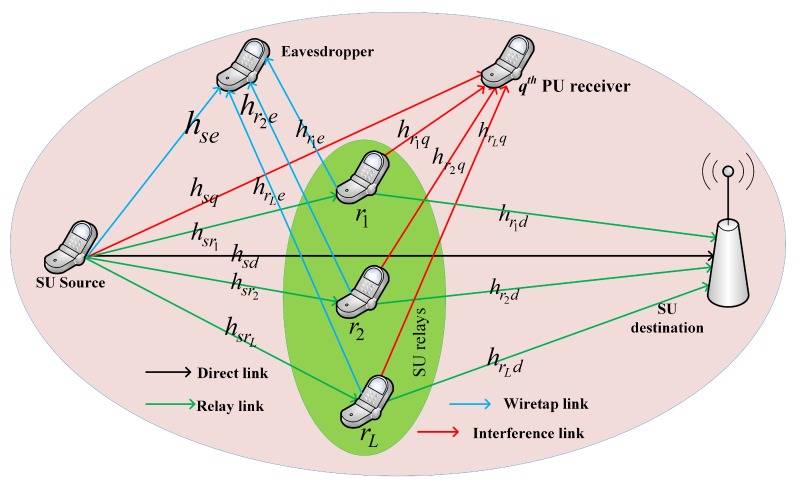
System model.

Consider a collaborative AF-based CCRNs in which the transmission power of the SUs are limited. As shown in Figure 1, a SU source *s* wants to transmit confidential data to the SU destination *d* with the aid of the selected SU relays $r_{j}$, where j=1,2,3,…,L while an eavesdropper *e* (which is also a SU in the networks) attempts to intercept the transmissions. The PU receiver is labeled as the *q*-th PU q=1. The channel gains between the SU source *s* and the destination *d* and the eavesdropper *e* are denoted by $h_{sd}$ and $h_{se}$, respectively. The SU relays corresponding channel gains between the SU destination *d* and the eavesdropper *e* are denoted as $h_{r_{j}d}$ and $h_{r_{j}e}$, respectively. In an addition, the corresponding interference channel gain (ICG) between the SU source *s* and the PU receiver *q* and the ICG between the SU relays $r_{j}$ and the PU receiver *q* are also denoted as $h_{sq}$ and $h_{r_{j}q}$, respectively.

In the considered system model, the SU utilizes the spectrum of the PU under its tolerable interference. Due to the limitation of transmission power, efficient PA may be considered in system design to guarantee the efficient utilization of the limited power to protect the interference to the PU receiver. Each user with a single antenna is operated in a half-duplex mode. The SU destination and the eavesdropper employ a maximum ratio combining (MRC) technique to maximize their secrecy capacity. The transmission is thus completed in two phases. In the first transmission phase, the SU source broadcasts data symbol $x_{s}$ to the SU relays and the SU destination. Then, the SU relays amplify the received symbol of the SU source and retransmit it to the SU destination in the second transmission phase. The eavesdropper can overhear both phases of the transmissions because the eavesdropper is also a secondary user. We assume that the SU relays forward the amplified version of the received signal of the SU source in a preassigned orthogonal channel. In a similar fashion as in [11,12,13,18,26], we assume that the perfect CSI about all the channels is available at the receiver for prior transmission.

### Problem Formulation

We denote $h_{sd}$ and $h_{se}$ as the channel gain between the SU source and the SU destination and the channel gain between the SU source and the eavesdropper, respectively. Channel gain between the SU source and the *j*-th SU relay is denoted as $h_{sr_{j}}$, where the set of SU relays is denoted by j=1,2,3,…,L. Let $h_{r_{j}d}$ and $h_{r_{j}e}$ stand for the channel gain between the *j*-th SU relay and the SU destination and the channel gain between the *j*-th SU relay and the eavesdropper *e*, respectively. In the first transmission phase, the received signal at the SU destination, the *j*-th SU relay and the eavesdropper are expressed as

(1) $$y_{sd}=\sqrt{P_{s}}h_{sd}x_{s}+n_{sd}$$

(2) $$y_{sr_{j}}=\sqrt{P_{s}}h_{sr_{j}}x_{s}+n_{sr_{j}}$$

(3) $$y_{se}=\sqrt{P_{s}}h_{se}x_{s}+n_{se}$$

where $P_{s}$ is transmission power of the SU source, with $n_{sd}$, $n_{sr_{j}}$, and $n_{se}$ are being the AWGN of the related channels, respectively. In this paper, it is assumed that the AWGN is independent for all channels and it follows same distributions of zero mean and variance $\sigma_{n}^{2}$. During the second transmission phase, each SU relay amplifies received signal $y_{sr_{j}}$ by amplification gain $g_{r_{j}}$ and broadcasts the amplified signal for the SU destination. The eavesdropper in the network can also overhear the information from the *j*-th SU relay due to the nature of wireless communications medium. Therefore, the received signals at the *j*-th SU relay and the eavesdropper can be respectively expressed as

(4) $$\begin{array}{c} y_{r_{j}d}=y_{sr_{j}}h_{r_{j}d}g_{r_{j}}+n_{r_{j}d}\\ \;\;\;\;\;\;\;\;\;\;\;\;=\;\sqrt{P_{s}}g_{r_{j}}h_{r_{j}d}h_{sr_{j}}x_{s}+g_{r_{j}}h_{r_{j}d}n_{sd}+n_{r_{j}d} \end{array}$$

(5) $$\begin{array}{c} y_{r_{j}e}=y_{sr_{j}}h_{r_{j}e}g_{r_{j}}+n_{r_{j}e}\\ \;\;\;\;\;\;\;\;\;\;\;\;=\sqrt{P_{s}}g_{r_{j}}h_{r_{j}d}h_{sr_{j}}x_{s}+g_{r_{j}}h_{r_{j}e}n_{r_{j}d}+n_{r_{j}e} \end{array}$$

where $n_{r_{j}d}$ and $n_{r_{j}e}$ are the AWGN related to their respective channels. The amplification gain of the *j*-th SU relay is defined in [5] as follows

(6) $$g_{r_{j}}=\sqrt{\frac{P_{r_{j}}}{{\left|y_{sr_{j}}\right|}^{2}}}\;=\;\sqrt{\frac{P_{r_{j}}}{P_{s}{\left|h_{sr_{j}}\right|}^{2}+\sigma_{n}^{2}}}\;$$

where $P_{r_{j}}$ denotes the transmission power of the *j*-th SU relay. By replacing Equation (6) into Equations (4) and (5), the signal-to-noise ratio (SNR) of the channels related to the $h_{r_{j}d}$ and $h_{r_{j}e}$ can be respectively, obtained by [12] as follows

(7) $$\gamma_{r_{j}d}=\frac{m_{r_{j}}v_{r_{j}}P_{s}P_{r_{j}}}{1+m_{r_{j}}P_{s}+v_{r_{j}}P_{r_{j}}}$$

(8) $$\gamma_{r_{j}e}=\frac{m_{r_{j}}u_{r_{j}}P_{s}P_{r_{j}}}{1+m_{r_{j}}P_{s}+u_{r_{j}}P_{r_{j}}}$$

where $m_{r_{j}}=\frac{{\left|h_{sr_{j}}\right|}^{2}}{\sigma_{n}^{2}}$, $v_{r_{j}}=\frac{{\left|h_{r_{j}d}\right|}^{2}}{\sigma_{n}^{2}}$, and $u_{r_{j}}=\frac{{\left|h_{r_{j}e}\right|}^{2}}{\sigma_{n}^{2}}$ for all j=1,2,3,…,L. In this paper, we define a binary variable $\varepsilon_{r_{j}}$ that indicates the system decision whether the *j*-th SU relay is selected for signal forwarding or not. Therefore, the binary variable is expressed as

(9) $$\varepsilon_{r_{j}}=\left\{\begin{array}{c} 1\;\;\;\;\;\;\;\;\;\;\mathrm{if}\;\mathrm{the}\;\;j-\mathrm{th}\;\;\mathrm{SU}\;\mathrm{relay}\;\mathrm{is}\;\mathrm{selected}\\ 0\;\;\;\;\;\;\;\;\;\mathrm{otherwise} \end{array}\right.$$

In this paper, MRC technique is used to maximize $\gamma_{r_{j}d}$ and $\gamma_{r_{j}e}$, respectively. Therefore, the data rate at the SU destination, $R_{d}$ and the eavesdropper, $R_{e}$ can be expressed as

(10) $$R_{d}=\frac{1}{2}\log2\left(1+\frac{P_{s}{\left|h_{sd}\right|}^{2}}{\sigma_{n}^{2}}+\sum_{j=1}^{L}\varepsilon_{r_{j}}\gamma_{r_{j}d}\right)$$

(11) $$R_{e}=\frac{1}{2}\log2\left(1+\frac{P_{s}{\left|h_{se}\right|}^{2}}{\sigma_{n}^{2}}+\sum_{j=1}^{L}\varepsilon_{r_{j}}\gamma_{r_{j}e}\right)$$

The SR, $R_{\sec}$ is defined as [2]

(12) $$R_{\sec}={\left[R_{d}-R_{e}\right]}^{+}$$

In transmission-power and interference-limited CCRNs, we need to transmit information as much as possible by utilizing available transmission power while satisfying the interference requirements of the PU receiver. Moreover, the SR of the networks is also needed to be maximized, and the SR maximization optimization problem of the considered system model can be formulated as

(13) $$\max_{P_{s},P_{r_{j}},\;\varepsilon_{r_{j}}}\;\;\;R_{\sec}$$

subjected to

(14) $$C.1:\;\;\;\;\;0\leq P_{s}\leq P_{SU}^{Max}$$

(15) $$C.2:\;\;\;\;\;0\leq P_{r_{j}}\leq P_{SU}^{Max}\;\;\;\;\;\forall j=1,2,3,\ldots ,L$$

(16) $$C.3:\;\;P_{s}{\left|h_{sq}\right|}^{2}\leq I_{Max}$$

(17) $$C.4:\;\;\;\;\;\sum_{j=1}^{L}\varepsilon_{r_{j}}P_{r_{j}}{\left|h_{r_{j}q}\right|}^{2}\leq I_{PU}^{Max}$$

(18) $$C.5:\;\;\;\;\;\varepsilon_{r_{j}}\in \left\{0,1\right\}\;\;\;\;\;\forall j=1,2,3,\ldots ,L$$

where $P_{SU}^{Max}$ and $I_{PU}^{Max}$ are the maximum permissible transmission power of the SUs, and acceptable interference level of the PU receiver, respectively. The C.1 and C.2 are the transmission power constraints of the SU source and the SU relay, respectively, while the C.3 and C.4 represent the interference constraints of the PU receiver during first and second transmission phases of the networks, respectively.

## 4. The Proposed GA-based MRS and PA Scheme for CCRNs

Our objective in this paper is to maximize the SR of CCRNs as defined in Equation (12) by satisfying transmission power and the interference requirements of the networks. As is known, the PA at the SU source depends mainly on two constraints: transmission power and the interference constraints as described in Equations (14) and (16). The optimal transmission power of the SU source is defined in [19] as follows

(19) $$P_{s}^{*}=\min\left\{\left(P_{SU}^{\max},\frac{I_{PU}^{Max}}{{\left|h_{sq}\right|}^{2}}\right)\right\}$$

In the second transmission phase, we need to select the SU relays and assign power to those selected SU relays to maximize the SR of the network without generating any harmful interference to the PU receiver. The optimization problem during second transmission phase can be further formulated as

(20) $$\max_{P_{r_{j}},\;\varepsilon_{r_{j}}}\;\;\;R_{\sec}\;\mathrm{subject}\mathrm{to}C.2,C.4,\mathrm{and}C.5$$

The optimization problem in Equation (20) is a MIP problem, where SU RS indicator $\varepsilon_{r_{j}}$ and SU relay PA $P_{r_{j}}$ are a binary integer variable and a real valued parameter, respectively. In this paper, a GA-based scheme is proposed to solve the MIP problem. The proposed scheme divides each chromosome into two parts (SU relays selection and PA to the selected SU relays). The genes in the SU relays selection and the PA to those selected SU relays are consist of binary integer strings and a sequence of real numbers, respectively. In the proposed scheme, the genes with having maximum fitness value will be selected as the best SU relays and optimal assignment of transmission power to the selected SU relays to forward the SU source information to the SU destination.

**Figure 2:**
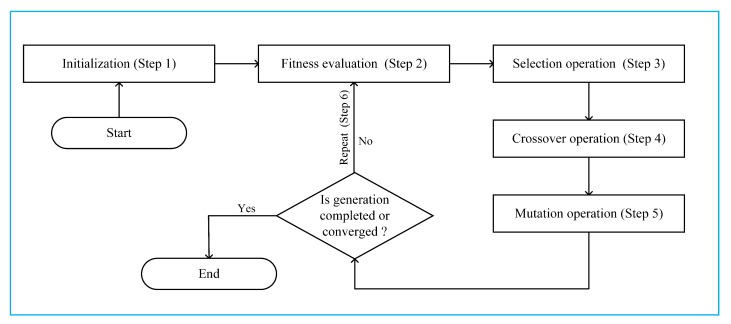
Flowchart of the proposed GA.

To solve the optimization problem in Equation (20), in the paper we adopt a general GA which includes the initialization of the population, evaluation of fitness function, and some genetic operations, such as the selection, crossover, and mutation [27,28,29]. The GA also requires a repeated iteration process until a near-optimal solution is obtained by the algorithm [30,31]. A pseudo-code of the GA can be given as in Algorithm 1 [31]. Based on the GA, we propose the GA-based MRS and PA scheme for SR maximization. The Figure 2 shows the flowchart of the proposed scheme, which consists of 6 steps as follows:

- Step 1 (Initialization): Randomly create populations for all chromosomes $\forall_{c}=1,2,3,\ldots ,T$.
- Step 2 (Evaluation): Calculate the fitness value of each generation of the proposed scheme, and normalize the transmission power of the SU relays to adhere to the constraints in Equations (15) and (17).
- Step 3 (Selection operation): In the proposed scheme, we use a roulette wheel selection method to breed a new generation to save the best δ chromosome.
- Step 4 (Crossover operation): Repeat the crossover operation to generate a new population set with a crossover probability $P_{c}$.
- Step 5 (Mutation operation) : Repeat the mutation operation to generate a new population set $P_{m}$.
- Step 6 (Repeat): The steps of the proposed scheme will be repeated for the next generation until the generation is completed or converged.

In the next sub-section, we will give more detailed descriptions on the implementation of each step.

**Algorithm 1:** Pseudocode of the GA.1 Choose an initial random population of individuals 2 Evaluate the fitness of the individuals 3 **Repeat** 4 **Selection operation** 5 *// Select the best individuals by roulette wheel selection* 6 *z*:= randomnumber, where 0≤z≤1 7 *sum*:= 0; 8 **for** each chromosome δ 9 **Calculate**
$P_{\delta }=\frac{f\left(\delta \right)}{\sum_{M=1}^{T}f\left(\delta_{M}\right)}$ 10 **Calculate**
$sum:=sum+P_{\delta }$ 11 **if**
z<sum 12 **Return**
δ 13 **end if** 14 **end for** 15 **Crossover operation** 16 Generate new individuals by using crossover operation of the GA with a crossover probability $P_{c}$ 17 **Mutation operation** 18 Mutate the generated offspring with a mutation probability $P_{m}$ 19 **Evaluate** the fitness of the new individuals 20 **Replace** the *worst* individuals of the population by the new individuals 21 **Until** the stopping criteria met


### 4.1. Steps of the Proposed GA-Based MRS and PA Scheme for SR Maximization

#### 4.1.1. Step 1: Initialization of the Population

The SU relays selection is comprised of *T* parent chromosomes, where *T* is the number of populations. The SU relays selection is comprised of $\left|j\right|$ genes and each of the genes is randomly distributed $\left\{1,2,3,\ldots ,\left|j\right|\right\}$. The chromosome structure in the SU relays PA is also comprised of $\left|j\right|$ genes which are randomly assigned between zero and the maximum admissible transmission power of each SU relay. The admissible transmission power of the *j*-th SU relay can be calculated as

(21) $$P_{r_{j}}^{Max}=\frac{I_{PU}^{Max}}{{\left|h_{r_{j}q}\right|}^{2}}$$

#### 4.1.2. Step 2: Evaluation

In each generation of the proposed scheme, the fitness value is calculated by substituting $\varepsilon_{r_{j}}$ and $P_{r_{j}}$ into Equation (20). Meanwhile, we also normalize the transmission power of the SU relay in this step to satisfy the constraints of Equations (15) and (17). If the constraints are not satisfied, the chromosome will be discarded for the step 3. The normalized transmission power of the SU relay can be determined as

(22) $$P_{r_{j}}^{Norm}=P_{r_{j}}^{Max}\times \frac{P_{r_{j}}}{\sum_{k=1}^{L}P_{r_{k}}}$$

#### 4.1.3. Step 3: Selection Operation

To achieve a better chromosome (solution) or survivor selection, roulette wheel selection is applied to breed a new generation. In roulette wheel selection, the probability that chromosome δ is chosen can be computed as

(23) $$P_{\delta }=\frac{f\left(\delta \right)}{\sum_{M=1}^{T}f\left(\delta_{M}\right)}$$

where $f\left(\delta \right)$ is the fitness value of the chromosome δ. In the proposed scheme, the chromosomes with a lower fitness value (a lower SR) will be discarded, but those with a higher fitness value will survive as parents to generate new offspring.

#### 4.1.4. Step 4: Crossover Operation

In the proposed scheme, a crossover generates new offspring by exchanging genes between two parent chromosomes. The crossover between good parents generates well-performing children, or even better ones. Thus, the parents selected through roulette wheel selection method are used for the crossover to produce offspring with crossover probability $P_{c}$.

#### 4.1.5. Step 5: Mutation Operation

The mutation operation of the proposed scheme is separated into two parts: integer number mutation for the SU relays selection and the real valued mutation for the SU relays PA. In the mutation step, the generated offspring from the crossover step will be considered for mutation, and the neighboring bits are randomly selected and exchanged with each other, i.e., 1 to 0 or 0 to 1 with mutation probability $P_{m}$. The offspring then form new population and the fitness of its chromosome will be evaluated before the next evaluation.

#### 4.1.6. Step 6: Repeat

The GA repeats from evaluation step to mutation step until it meets the maximum number generations $I_{g}$. The chromosome which have maximum fitness value (SR) will be chosen. However, utmost number of genes may not satisfy the constraint in Equation (17), and thus, the SR of those corresponding genes can be zero. In high SNR region, obtaining proper combinations of the genes to fit the constraint is also a challenging task. Therefore, the best string will be selected based on an additional fitness value. The additional fitness value $D^{\left(c\right)}$ for each chromosome can be given as

(24) $$D^{\left(c\right)}=∥\sum_{j=1}^{L}\varepsilon_{r_{j}}P_{r_{j}}{\left|h_{r_{j}q}\right|}^{2}-I_{PU}^{Max}∥.$$

Indeed, the best chromosome in this case is the one that provides the lowest $D^{\left(c\right)}$. The gene in SU relays selection and SU relays PA with the maximum fitness value of the chromosome will be selected to transmit information to the SU destination. The proposed GA-based MRS and PA scheme for maximizing the SR of CCRNs is detailed in Algorithm 2.

**Algorithm 2:** The proposed GA-based MRS and PA scheme for SR maximization.1 **Input:**
$I_{PU}^{Max}$, $P_{SU}^{Max}$, $\sigma_{n}^{2}$, *L*, $h_{sd}$, $h_{se}$, $h_{sr_{j}}$, $h_{r_{j}d}$, $h_{r_{j}e}$, $h_{sq}$, $h_{r_{j}q}$, $I_{g}$, *T*, $P_{c}$, and $P_{m}$ 2 **Initialization:** 3 Set, $R_{\sec}^{\left(c\right)}=0$; where $c=\left\{1,2,3,\ldots ,T\right\}$ 4 **GA Initialization** (Step 1) 5 Randomly select $\varepsilon_{r_{j}}$ and allocate power $P_{r_{j}}$ to the SU relays 6 **while**
$I_{gen}\leq I_{g}$ or not converged **do** 7 Increase generation counter $I_{gen}=I_{gen}+1$ 8 **for**
c=1 to *T* 9 **Calculate**
$D^{\left(c\right)}=∥\sum_{j=1}^{L}\varepsilon_{r_{j}}P_{r_{j}}{\left|h_{r_{j}q}\right|}^{2}-I_{PU}^{Max}∥$ 10 **Calculate**
$\gamma_{r_{j}d}$ and $\gamma_{r_{j}e}$ by using Equations (7) and (8), optimal transmission power of the SU source $P_{s}^{*}$ from Equation (19), and $P_{r_{j}}$ from step 1 of the proposed scheme 11 **Calculate**
$R_{d}$ and $R_{e}$ by using Equations (10) and (11) and $\varepsilon_{r_{j}}$ from step 1 of the proposed scheme 12 **Evaluation** (Step 2) 13 **if** the constraints of C.1 (in Equation (15)) and C.4 (in Equation (17)) are satisfied 14 **Calculate** SR by substituting $\varepsilon_{r_{j}}$ and $P_{r_{j}}$ in Equation (20) 15 **else** 16 Secrecy rate $R_{\sec}^{\left(c\right)}=0$ 17 **end if** 18 **end for** 19 **Selection operation** (Step 3) 20 Select the best individuals to breed a new generation 21 **Crossover operation** (Step 4) 22 Perform crossover to produce new offspring with $P_{c}$ 23 **Mutation operation** (Step 5) 24 Mutate the resulting new offspring with $P_{m}$ 25 **end while** 26 **Return** the best solution of the problem in Equation (20)


## 5. Simulation Results

In this section, we present the simulation results to validate the performance of the proposed scheme. In this paper, we assume that the channel between any transmitter and the receiver follows by an independent complex Gaussian random variables with zero mean and variances of $\sigma_{ab}^{2}$, respectively. The variances of the corresponding channels are defined in [33] as

(25) $$\sigma_{ab}^{2}={\left(\frac{\lambda }{4\;d_{ab}\pi }\right)}^{\alpha }=\;{\left(\frac{c}{4\;d_{ab}f_{c}\;\pi }\right)}^{\alpha }$$

where $d_{ab}$ denotes the distance between the transmitter and the receiver, the wavelength is $\lambda =\left(\frac{c}{f_{c}}\right)$, where *c*, $f_{c}$, and α are the speed of light, the carrier frequency, and the path-loss exponent, respectively. The channel gain between any transmitter *a* and the receiver *b* thus, expressed as: $h_{ab}∼CN\left(0,\sigma_{ab}^{2}={\left(\frac{c}{4d_{ab}f_{c}\pi }\right)}^{\alpha }\right)$. The complex channel gain vector can be expressed as

(26) $$h_{ab}=h_{real}+j\times h_{ima}.$$

(27) $$h_{real}=\sigma_{ab}^{2}\times randn\;\left(1,1\right).$$

(28) $$h_{ima}=\sigma_{ab}^{2}\times randn\left(1,1\right).$$

The *randn* function generates arrays of random numbers whose elements are normally distributed with mean 0 and variance 1 in the Matlab. Finally, to get each channel gain we take absolute of $h_{ab}$, $\left|h_{ab}\right|$ which has Rayleigh distribution with the variance $\sigma_{ab}^{2}$. Therefore, in the paper we consider Rayleigh fading channel, which is considered as a statistical model for the effect of a propagation environment on a radio signal. Also, it is noteworthy that the effect of the distance between nodes, and path loss exponent on the channel gain is considered in terms of variance $\sigma_{ab}^{2}={\left(\frac{c}{4d_{ab}f_{c}\pi }\right)}^{\alpha }$. It means that each channel between two nodes will have Rayleigh channel of which the mean will mainly depend on the distance between nodes and path loss exponent. In this paper, we have simulated a static network where locations of the users are fixed but the CSI’s of the corresponding channel is slightly changed due to the complex Gaussian random variable. However, generation of more realistic network scenario with different channel distributions for mobility-aware CCRNs is left for our future work.

We compare the SR performance of the GA-based MRS and PA scheme (denoted as Proposed scheme) with following schemes: (1) Exhaustive search scheme [15] which is an advance scheme published recently to solve the RS and PA problems; (2) OpportunisticRS scheme [34,35] select the SU relays based on considering two-hop channel gains; (3) PartialRS scheme: select the SU relays based on considering one-hop channel gain; (4) RandomRs scheme: select the SU relays randomly. The RS rules of OpportunisticRS and PartialRS schemes are defined in [34,35] as follows

(29) $$r_{j}^{ORS}=\underset{r_{j}\in \;\;\left(j=1,2,3,\ldots ,L\right)}{arg\;max\;min}\left\{{\left|h_{sr_{j}}\right|}^{2},{\left|h_{r_{j}d}\right|}^{2}\right\}$$

(30) $$r_{j}^{PRS}=\underset{r_{j}\in \;\;\left(j=1,2,3,\ldots ,L\right)}{arg\;max}\left\{{\left|h_{sr_{j}}\right|}^{2}\right\}$$

We compare the performance of the OpportunisticRS scheme, the PartialRS scheme, and the RandomRS scheme by considering the equal power allocation (EPA) concept as used by Lu et al. [36]. The parameters used for simulations are summarized in Table 1. We use MATLAB software as a simulation tool to collect the simulation results of our proposed scheme and other comparison schemes. The computer which is employed for collecting simulation results had an Intel(R) Core(TM) i7-6700K central processing unit with random access memory of 16 GB. In MATLAB, we firstly set the network parameters of the considered system model and the parameters of the GA and then, we implement Algorithm 2 to get the simulation results. The following simulations results are obtained by averaging Monte Carlo trials. In each trial, the channel conditions are independently determined. The cases of performance analysis over α fading channels [5], different time-varying fading channel [37], different outdated channels can be tested with additionally studying the channel estimation, the RS, and the PA problems of CCRNs but it is out of scope in this paper.

### 5.1. SR Performance and Convergence of the Proposed Scheme

**Figure 3:**
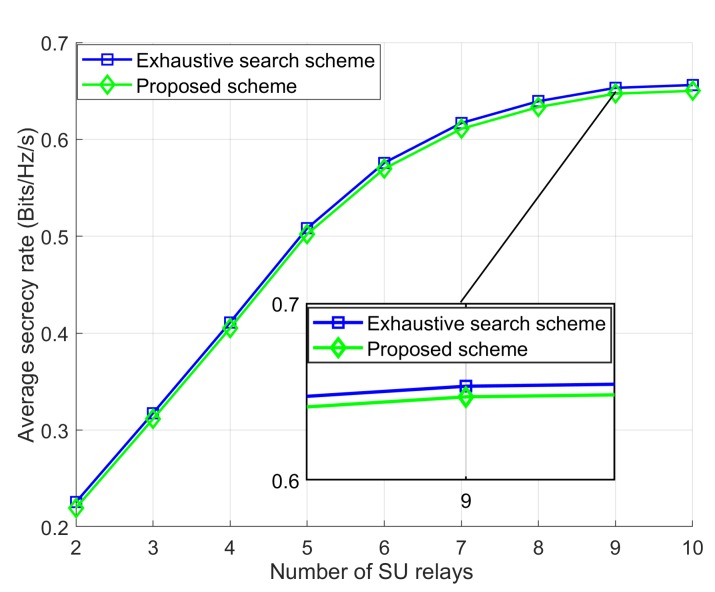
Average SR of the proposed scheme compared with the exhaustive search scheme.

**Figure 4:**
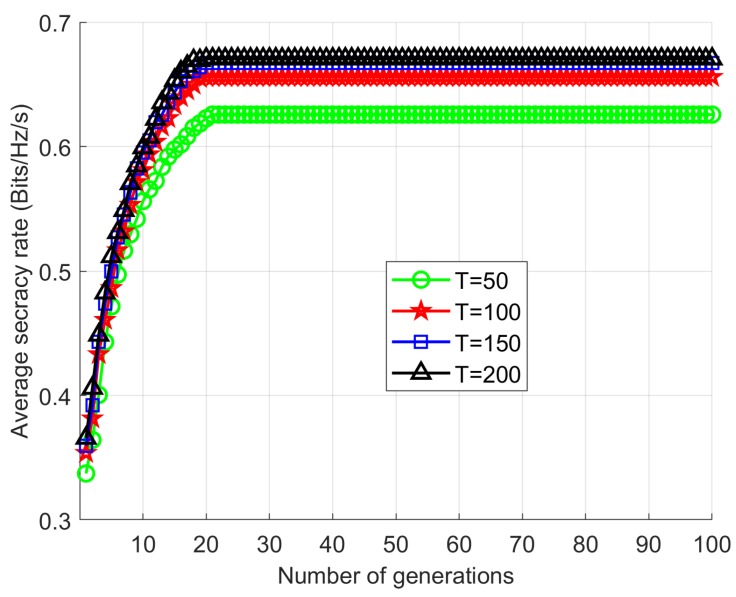
SR against the number of generations $I_{g}$.

To evaluate the SR performance and to show the proof of convergence our proposed scheme, we compare our Proposed scheme with the Exhaustive search scheme [9]. In the simulation, the location of the eavesdropper is fixed in one position when the values of $P_{SU}^{\max}$ and $I_{PU}^{\max}$ are set as 0 dBm and 5 dBm. In addition, the maximum number of generations and the number of populations are considered as $I_{g}=100$ , and T=100. We set the gap size as $10^{-4}$ for Exhaustive search scheme and searching for the optimal transmission power of the SU relays to maximize the SR of the networks. The simulation result is shown in Figure 3. We can observe that there is a negligible performance gap generated by the Proposed scheme and Exhaustive search scheme. However, the computational complexity of the Proposed scheme in terms of CPU time, which is shown in Table 2 is much lower than the Exhaustive search scheme. In Figure 3, we present the convergence of the fitness value (SR) of our Proposed scheme, where $P_{SU}^{\max}=0$ dBm, $I_{PU}^{\max}=5$ dBm, and L=10. As can be observe in Figure 4, the SR performance of the Proposed scheme is increased as the number of generations or the population size is increased. It can be well noted that Proposed scheme converges when the number of generation is approximately $I_{g}\leq \;20$.

### 5.2. SR Performance with Number of SU relays

**Figure 5:**
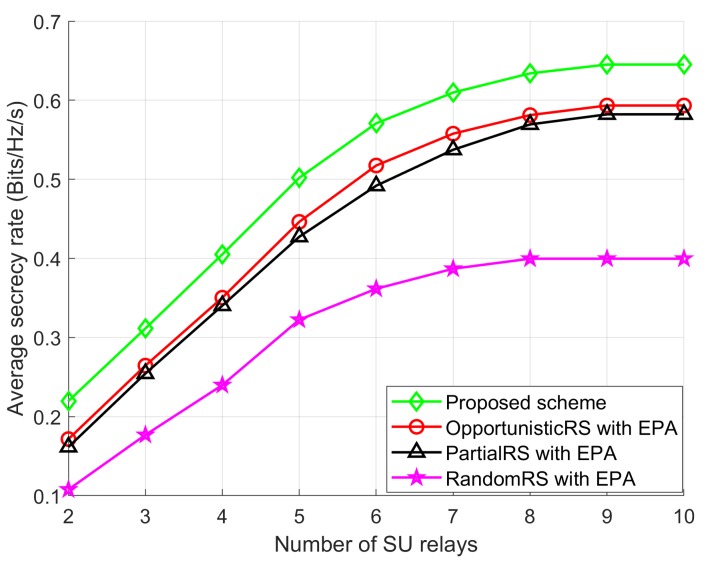
Average SR performance with *L*.

The average SR performance of the Proposed scheme and other schemes is illustrated in Figure 5 for different number of SU relays when $P_{SU}^{\max}$, $I_{PU}^{\max}$, $I_{g}$, and *T* are 0 dBm, 5 dBm, 100, and 100, respectively. As shown in Figure 5, the Proposed scheme shows better SR performance than the other schemes. With a large number of SU relays, the SR performance becomes flat because of the transmission power and interference constraints of the networks. Also, we observe that the Proposed scheme achieves a significant improvement in SR performance over other three schemes with EPA. Furthermore, Figure 5 reveals that the OpportunisticRS scheme outperforms the PartialRS scheme because of considering two-hop channel gains of the networks. The RandomRS scheme does not provide significant SR because it selects SU relays randomly in the environment.

### 5.3. SR Performance with Maximum Permissible Transmission Power

**Figure 6:**
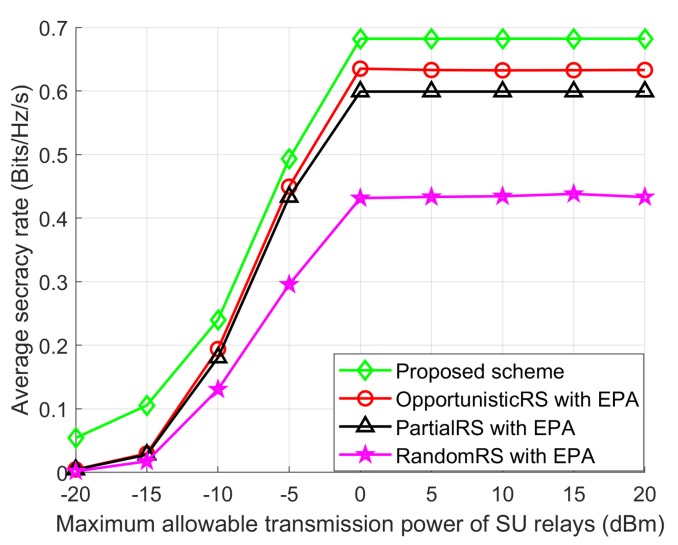
Average SR performance with $P_{SU}^{\max}$.

Figure 6 shows the average SR performance of the Proposed scheme with other schemes with changing the value of permissible transmission power of the SUs when $I_{PU}^{\max}\;$, $I_{g}$, *T*, and *L* are 5 dBm, 100, 100, and 10, respectively. The SR performance of all schemes increase as $P_{SU}^{\max}$ increases. When the $P_{SU}^{\max}$ is 0 dBm, the SR of all schemes become flat because the interference threshold of the PU receiver will not allow to assign more transmission power. As expected, the SR performance achieved by the Proposed scheme is much higher than that of the other schemes. Also, we can see that the Proposed scheme achieves a noteworthy improvement in SR performance over the other schemes with EPA. Due to the randomness in selecting the best SU relays, the RandomRS scheme with EPA shows lower average SR performance than that of OpportunisticRS and PartialRS schemes with EPA.

### 5.4. SR Performance with Acceptable Interference Threshold

**Figure 7:**
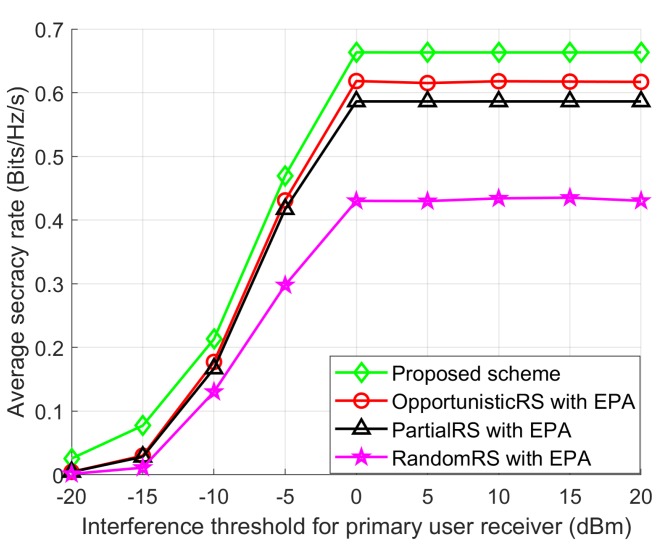
Average SR performance with $I_{PU}^{\max}\;$.

Figure 7 shows the average SR performance with the interference threshold for primary user receiver when $P_{SU}^{\max}$, $I_{g}$, *T*, and *L* and are 10, 0 dBm, 100, and 100, respectively. The average SR of all schemes increase as the interference threshold is increased. In the high interference threshold region, the SR is restricted because the transmission power constraints of the SUs play a dominant role, and the interference threshold has hardly any effect on the transmission power at the SU relays. As expected, the SR performance of the Proposed scheme is much higher than the other schemes. Also, we can see that the Proposed scheme achieves noteworthy improvement in SR performance over the other schemes. Due to the randomness in selecting the best SU relays, the RandomRS scheme provides a very low SR performance compared to other three schemes.

### 5.5. SR Performance with Changing the Distance of the Eavesdropper

**Figure 8:**
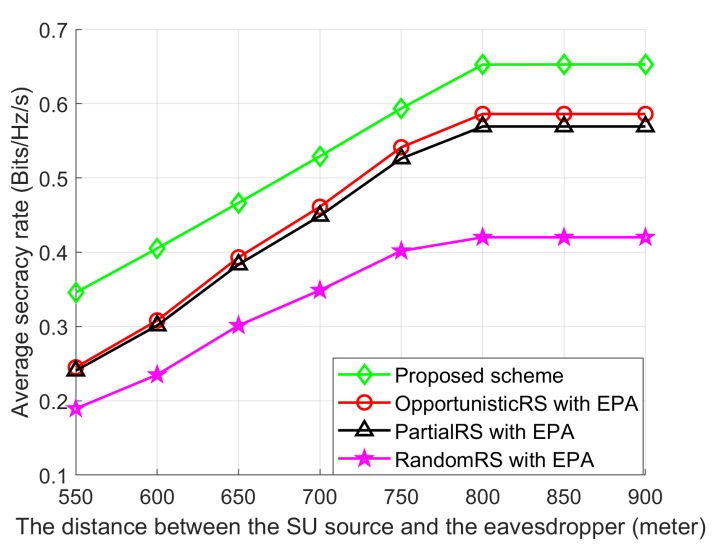
Average SR performance with $d_{SE}$.

The performance of the Proposed scheme, OpportunisticRS scheme, PartialRS scheme and RandomRS scheme over different locations of eavesdropper is shown in Figure 8 where $D_{sd}$ is fixed at 500 m and the $D_{se}$ is varied from 550 m to 900 m. We can observe that average SR performance of the Proposed scheme shows better performance than the other schemes. With the increasing of $D_{se}$, the SR performance of all schemes is also increasing because $h_{sd}$ and $h_{r_{j}d}$ are becoming strong gradually compared with $h_{se}$ and $h_{r_{j}e}$ due to the movement of the eavesdropper in the networks.

### 5.6. SR Performance with the Variance of the AWGN

**Figure 9:**
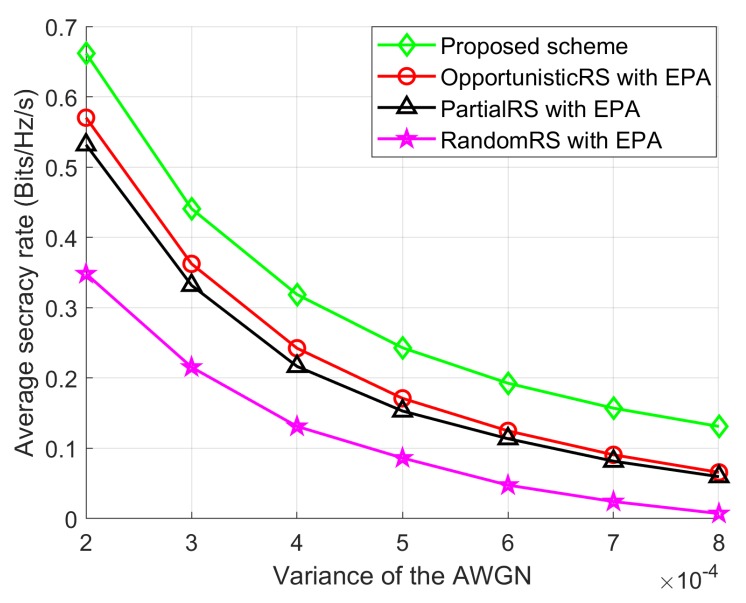
Average SR performance with $\sigma_{n}^{2}$.

We also compared the average SR against variances in AWGN, as shown in Figure 9 when $P_{SU}^{\max}$, $I_{PU}^{\max}$, $I_{g}$, *T*, and *L* are 0 dBm, 5 dBm, 100, 100, and 10, respectively. As can be seen in Figure 9, the performance of all schemes declines as the variance of AWGN increases. With an increasing of $\sigma_{n}^{2}$, the SNR of the corresponding communication links becomes weaker gradually, which can decrease $R_{d}$ and $R_{e}$. Also, note that average SR performance of the Proposed scheme is again better than other schemes. The RandomRS scheme shows lower performance because of the randomness in selecting the SU relays.

### 5.7. Computational Complexity Analysis

To assess the computational overhead, we calculate the complexity of the Proposed scheme with other schemes in terms of arithmetic operations and the CPU time required to solve the optimization problem [28]. To obtain the solution of SU relays selection and PA through the Exhaustive search scheme, it requires high computational complexity. In the Proposed scheme, the computational complexity is determined based on the number of populations. For each generation of the Proposed scheme, we needed to calculate the fitness value for *T* times and one for each chromosome. Therefore, the total arithmetic operations needed by the Proposed scheme is about $O\left(GT\right)$. A comparison of the complexity for all schemes is shown in Table 3. To reveal the advantages of our Proposed scheme more appropriately, a comparison of the CPU times for all the schemes is presented in Table 2. To demonstrate the performance of average CPU time, we utilize the same simulation parameters as used for Figure 4. We can see from Table 2 that the CPU time required by all schemes increases with an increase in the SU relays, and the CPU time of other schemes is much lower than the Exhaustive search scheme when the step size for discretizing the maximum transmission power of the SU relays for Exhaustive search scheme is considered as $10^{-4}$. The CPU time required by all schemes are high when the number of SU relays is large because as there are more variables involved in calculating the SR of the networks.

## 6. Conclusions

In this paper, a low-complexity GA-based scheme is proposed to enhance physical layer security of CCRNs when a single eavesdropper coexist in the networks. As we consider an MIP problem, and its complexity increases as the number of SU relays are increased; we propose a GA-based scheme to solve it. By MRS and PA through the GA, the SR of the network is maximized while maintaining the transmission power and the interference requirements of the networks. Simulation results indicate that the proposed scheme achieves the near-optimal SR performance of the Exhaustive search scheme and provides better SR performance than some conventional RS schemes. In this paper, it is shown that the proposed scheme can provide solutions for MRS and PA in the presence of single eavesdropper when the perfect CSI about all the channels are available at the receiver for prior transmission. Imperfect CSI, different time-varying channels, and outdated channels are beyond the scope of this paper. Therefore, future research will include RS and PA through learning algorithm for mobility-aware multi-tier heterogeneous networks when multiple eavesdropper, multiple legitimate sources and destinations are coexisted in the network. The study of imperfect CSI and channel estimation of different time-varying channels to maximize the SR of mobility-aware CCRNs are also remained as a future work.

## Figures and Tables

**Table 1:** Simulation parameters.

Name of the Parameter	Notation	Parameter Value
Network area		600 m × 600 m
Number of SU source	*s*	1
Number of SU relays	*L*	10
Number of SU destination	*d*	1
Number of eavesdropper	*e*	1
SU source coordinates		(0,0)
SU destination coordinates		(500,0)
Carrier frequency	$f_{c}$	700 MHz
Path-loss exponent [6]	α	4
Population of the GA	*T*	100
Number of generations	$I_{g}$	100
Crossover probability	$P_{c}$	0.6
Mutation probability	$P_{m}$	0.02

**Table 2:** CPU time against total number of SU relays.

*L*	ES Scheme	Proposed Scheme	OpportunisticRS Scheme	PartialRS Scheme	RandomRS Scheme
2	6.8517 (s)	2.2900 (s)	6.7500 (s)	6.9100 (s)	7.3100 (s)
4	300.6213 (s)	6.4500 (s)	21.7600 (s)	22.4900 (s)	22.0800 (s)
6	2172.5438 (s)	12.1200 (s)	38.2200 (s)	39.3500 (s)	38.4200 (s)
8	3277.6352 (s)	20.1300 (s)	59.0400 (s)	58.5700 (s)	60.2300 (s)
10	5289.5759 (s)	29.8000 (s)	81.0000 (s)	80.8500 (s)	83.1400 (s)

**Table 3:** Comparison of the complexity of various schemes.

Name of the Scheme	Arithmetic Operations Required
Exhaustive search scheme	$O\left(2^{\left|j\right|}\right)$
OpportunisticRS scheme	$O\left(2\left|j\right|\right)$
PartialRS scheme	$O\left(2\left|j\right|\right)$
RandomRs scheme	$O\left(2\left|j\right|\right)$
Proposed scheme	$O\left(GT\right)$
